# Enniatins and Beauvericin as Emerging Mycotoxins in the Context of Climate Change in Europe

**DOI:** 10.3390/toxins18050209

**Published:** 2026-04-30

**Authors:** Francesca De Battistis, Chiara Civitelli, Valentina Prota, Francesca Caloni, Alberto Mantovani, Olimpia Vincentini

**Affiliations:** 1 Department of Food Safety, Nutrition and Veterinary Public Health, Italian National Institute of Health, 00161 Rome, Italy; 2 Department of Environmental Science and Policy (ESP), Università degli Studi di Milano, 20133 Milan, Italy; 3 Study Centre KOS—Science, Art, Society, 00144 Rome, Italy

**Keywords:** climate change, Europe, emerging mycotoxins, enniatins, beauvericin, occurrence

## Abstract

Emerging mycotoxins are unregulated natural toxins, often detected in small-grain cereal crops. They are produced by various *Fusarium* molds and have been reported in surveys conducted across Europe. Many *Fusarium* species that produce mycotoxins thrive and exhibit greater pathogenicity under relatively warm and humid conditions. Environmental conditions that promote fungal growth often also enhance mycotoxin accumulation. Various abiotic factors influence both *Fusarium* growth and mycotoxin biosynthesis, and several studies have associated these environmental conditions with the occurrence of enniatins (ENNs) and beauvericin (BEA) in cereal crops. Ongoing climate change in Europe may further support the spread and development of *Fusarium* species, potentially increasing the production of emerging mycotoxins. Following recent updates on the occurrence of these mycotoxins, this review evaluates the scientific literature concerning *Fusarium* species responsible for ENNs and BEA production.

## 1. Introduction

One of the main environmental concerns of the 21st century is undoubtedly linked to climate change, which includes rising sea levels, reduced drinking water reserves, the expansion of desert areas, changes in rainfall patterns, and the intensification and increased frequency of extreme weather events, which pose many risks [1]. Higher temperatures, together with extreme rainfall or prolonged drought, cause greater stress on plants, making plant-based foods more vulnerable to fungal infections and enhancing the production of mycotoxin metabolites, including “modified” or “masked” forms [2], as well as of new and/or previously unrecognized “emerging mycotoxins.” In addition, niche transition is observed within mold communities, resulting from increased heat tolerance of toxigenic species and the emergence of new fungi resistant to high temperatures. Furthermore, climate change can activate previously silent (or hidden) secondary metabolic clusters, causing changes in the epigenetic mechanisms of fungi, generating differential expression of toxin-synthesizing genes and a consequent increase in exposure to mycotoxins [3]. Due to climate change, the presence of mycotoxins is expected to be influenced in Europe in the coming years. This includes well-established hazards such as aflatoxins or ochratoxins as well as emerging *Fusarium* mycotoxins such as enniatins (ENNs) and beauvericin (BEA) [4]. Emerging mycotoxins are fungal metabolites increasingly detected in crops used for feeds and foods, for which no regulatory limits exist; thus, they can be considered as “emerging risks” according to the definition of the European Food Safety Authority (EFSA): “a risk resulting from a newly identified hazard to which a significant exposure may occur” [5].

Several different crops, including barley, wheat, corn, peanuts, cotton and many others, are susceptible to infection by these fungal species. In particular, it is estimated that *Fusarium* mycotoxins are moving towards northern Europe, while Aspergillus species are concentrating mainly in southern and central Europe [4]. The increased use of alternative protein sources in place of fishmeal in aquaculture is also leading to an increased risk of mycotoxin contamination. There is growing concern about human exposure through consuming fish, as current data highlights that fish are exposed to common and emerging mycotoxins [6].

Fungal proliferation and mycotoxin production can also occur post-harvesting in environments with high temperature and moisture [3,4], and the risk of production can be increased by improper storage, transportation, poor harvesting and processing practices [7]. It is a topic that has attracted worldwide attention due to economic losses related to animal productivity, trade and human health [8]. Indeed, toxicological concerns have been identified for several emerging mycotoxins in both animals and humans [9,10].

This paper reviews and summarizes evidence about the toxicological effects and occurrence of emerging unregulated, contaminants of multiple commodities, specifically enniatins and beauvericin, highlighting in particular their current relevance in the European scenario.

Ultimately, this review underscores the pressing need to redefine the mycotoxin risk framework and develop mechanistic information prediction tools tailored to climate change, thereby warning of new risks associated with global warming.

## 2. Emerging Mycotoxins: Characteristics and Toxicological Implications

Enniatins and beauvericin have very similar structures; both are cyclic hexadepsipeptides, that is, molecules composed of six alternating units of:

- Three residues of D-2-hydroxyisovaleric acid (an α-hydroxycarboxylic acid),
- Three residues of N-methylated amino acids.

**Figure 1:**
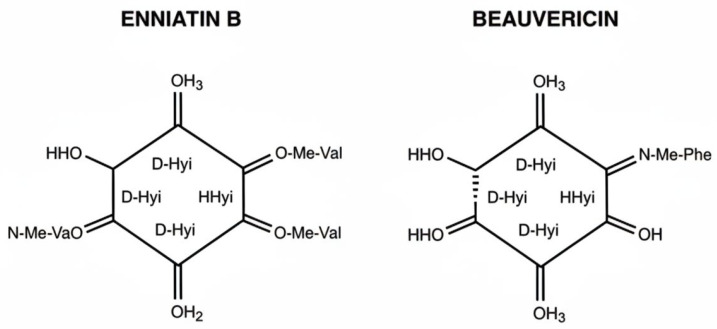
Molecular structures of enniatin B and beauvericin.

These subunits alternate and are connected through peptide and ester (lactone) bonds, forming a closed cyclic ring (Figure 1 and Table 1).

### 2.1. Enniatins

Enniatins (ENNs) are cyclohexapeptides composed of alternating residues of three N-methyl amino acids, generally valine, leucine and isoleucine, and three hydroxy acids such as hydroxyisovaleric acid. ENNs A, A1, B and B1 are the most widespread in Europe and are mainly present as natural contaminants in cereals [11]. Due to their lipophilic properties, ENNs are incorporated into the lipid bilayers of cell membranes and, following the formation of cation-selective pores, an increase in cation permeability is achieved, resulting in an alteration of the physiological level of cations in the cell [12]. ENNs exhibit insecticidal, antifungal, antibacterial and anthelmintic activity; they have also demonstrated cytotoxicity in vitro, although some in vivo studies have not shown toxicity [1,2,3,4,5,6,7,8,9,10,11,12,13,14,15,16,17].

ENNs exert toxicological different mechanisms of action, and the synergic and antagonistic activity within them and in relation to conventional mycotoxins, still under investigation, represent a problem of considerable interest, due to the complex toxicological implications [18,19,20,21]. The available data indicate that cereals are the crops most contaminated by ENNs. Detectable residues of ENNs were observed in products derived from cereals, such as flours (7.8%), pasta (6.30%), or couscous (1.42%); due to their widespread presence and the lack of official control programs, the potential impact of ENNs in Europe might be underestimated [9]. ENNs were also detected in other commodities, like baby food products, spices, and nutritional supplements. Furthermore, ENNs’ chemical structure does not appear to be altered by certain food processes, including cooking, baking, frying, roasting, etc. [22,23].

#### 2.1.1. Enniatin A

Enniatin A (ENN A) is a *Fusarium* mycotoxin commonly found in cereals, particularly corn, resulting in significant product losses [24]. Most data are currently available for BEA, ENN B and ENN B1, while ENN A and ENN A1 are among the least studied mycotoxins. In fact, EFSA has identified a critical gap in its scientific opinion as being the lack of toxicokinetic data [25], and several studies have been conducted that have provided new information.

In an in vitro study, ENN A showed concentration and time-dependent cytotoxic effects in HeLa cells, with IC_50_ values of 1.15 μM after 24 h and 0.57 μM after 48 h. In human lymphocytes, ENN A did not significantly induce chromosome aberrations, sister chromatid exchanges, or micronuclei, except for a slight increase in SCEs at 0.57 μM after 48 h. Replication and nuclear division indices were not affected, while the mitotic index was significantly reduced at most concentrations. Toxic effects were observed in lymphocytes at concentrations above 2.29 μM. Furthermore, ENN A significantly increased DNA damage, as indicated by increased comet tail intensity, at most tested concentrations [26].

In an in vitro study using Caco-2 cells, about 20% of the compound was transported from the apical to the basolateral side after 1 h, rising to 77% after 4 h of exposure to 1.5 μM ENN A, suggesting that ENN A exhibits greater transport efficiency than ENN B and ENN B1 [27]. Transport studies conducted on different human cell lines following treatment with an ENN mixture containing 3% ENN A revealed, via Liquid Chromatography–Tandem Mass Spectrometry (LC-MS/MS) analysis, that the toxin is a substrate of multidrug-resistant proteins, including P-glycoprotein (P-gp) [28].

In a 28-day in vivo study on five Wistar rats, the absorption and distribution of ENN A were analyzed following feeding with naturally contaminated feed containing 465 mg of ENN A/kg of feed. Significant levels of ENN A were detected throughout the gastrointestinal tract, namely 9.6 μg/g in the jejunum, 7.3 μg/g in the colon, 4.6 μg/g in the stomach, and 1.3 μg/g in the duodenum, suggesting incomplete absorption [15].

#### 2.1.2. Enniatin A1

Enniatin A1 (ENN A1) is tied to species whose distributions and toxin profiles are shifting with warming and altered precipitation patterns in Europe. Evidence links warmer, humid conditions and changes in rainfall to altered *Fusarium* communities and enniatin occurrence, with regional shifts already observed.

ENN A1 is produced by several *Fusarium* species that commonly infect small-grain cereals, and the composition of those species in Europe is changing with climate signals. Enniatin A1 is a significant emerging mycotoxin with widespread occurrence, particularly in cereals and baby food, produced by numerous *Fusarium* species. ENN A1 is a cyclohexadepsipeptide mycotoxin with an ionophoric nature [29]. The cyclic structure consists of a hexadepsipeptide core with variable R groups that define specific enniatin structures and is formed by incorporation of two isoleucine and one L-valine amino acid residues. It exhibits potent cytotoxicity through multiple mechanisms with ionophoric activity, disrupting cellular ionic homeostasis, enabling potent cytotoxic effects and acting as an endocrine disruptor, yet it remains unregulated despite these concerning characteristics [9].

Enniatins A1 and B1 are emerging mycotoxins that affect Ca^2+^ homeostasis through different mechanisms. ENN A1 induces Ca^2+^ entry via store-operated channels, an effect modulated by mitochondrial function. In contrast, ENN B1 acts on a different Ca^2+^ channel and is also influenced by mitochondrial activity. Both toxins impair mitochondrial function by inducing the opening of the mitochondrial permeability transition pore and do not act as Ca^2+^ ionophores [21].

Emerging research shows that ENN A1 can cause cytotoxic effects in cell-based in vitro studies. In the Neuroblastoma cell line SH-SY5Y after 24 h exposure, ENN A1 has been shown an IC_50_ of 2 μM, to induce apoptosis at 3.4 μM and to cause a 50% increase in Reactive Oxygen Species [ROS] production at 0.1–0.5 uM [19,20]. In Caco-2 intestinal epithelial cells, the IC_50_ values ranged from 7.7 μM after 48 h to 1.3 μM after 72 h of exposure. Among the tested compounds, ENN A1 exhibited the highest cytotoxicity in both HepG2 and HT29 cell lines, with IC_50_ values of 2.6 μM and 1.4 μM, respectively, following 48 h of treatment [30].

In vitro studies across diverse cell lines have reported pronounced, dose-dependent cytotoxic effects of ENNs; nevertheless, investigations into their stability during digestion remain limited. It has been found that food matrix and digestive conditions influence ENN absorption and distribution [31].

#### 2.1.3. Enniatin B

Enniatin B [ENN B] is produced by *Fusarium* fungi as a secondary metabolite and exhibits antibacterial, antifungal, anthelmintic, herbicidal, and insecticidal properties.

ENNB has been shown to act against a range of microorganisms, particularly Gram-positive bacteria like Staphylococcus aureus, Bacillus subtilis or Enterococcus faecalis. These are generally more susceptible because their cell wall structure is easier for ionophores like ENN B to disrupt.

However, it is also effective although with a minor sensitivity on Gram-negative bacteria such as Escherichia coli and Salmonella enterica.

ENNB can also inhibit fungal and yeast growth such as Candida albicans and Saccharomyces cerevisiae due to its membrane-disrupting action [29].

It is also detected, in association with other mycotoxins, as a contaminant in cereals and other food products, where it coexists with other mycotoxins [21]. Of all the ENNs, ENN B is currently the most common, as it is most prevalent in unprocessed and processed cereals from European countries.

It has been demonstrated that ENN B uses ionophore characteristics to form stable complexes with cations, causing alterations in intracellular ion concentration with a consequent increase in membrane permeability [32,33,34,35].

The oxidative stress associated with ENN B is caused by the production of ROS and lipid peroxidation [LPO] observed in vitro in Caco-2 cells [11,36]. In addition, ENN B has been shown to cause cell cycle impairment in both in vitro human cancer cell lines and in vivo [37,38]. Several studies show that the alkaline comet assay is negative in V79 and Caco-2 cells following administration of ENN B concentrations ranging from 1 to 100 μM; therefore, ENN B does not cause any DNA damage, despite its high cytotoxic potential [36].

Many studies have revealed that ENN B, alone or in combination with other mycotoxins, induces the formation of apoptotic bodies and nuclear fragmentation [36,37,38,39,40,41,42,43]. Enniatin B exhibits biological activity by inhibiting acyl-CoA:cholesterol acyltransferase (ACAT) activity, an effect that may be relevant for the prevention and treatment of atherosclerosis and hypercholesterolemia.

It has been shown that ENN B and BEA inhibit the transport of cathepsin B from the Golgi apparatus to the lysosomes following an increase in lysosomal pH, thereby causing the release of cathepsin B into the cytosol, which activates caspases and thus the apoptotic pathway [44].

#### 2.1.4. Enniatin B1

Enniatin B1 (ENN B1) is less studied and investigated than other ENNs but results as one of the relevant and often prevalent contaminants in commodities among ENNs [45,46,47,48,49], recently detected in infant foods, where ENN B1 was one of the main detected mycotoxins, although the calculated risk was considered negligible [50], and in cheese, suggesting that some *Penicillium* species, used in cheese making, are ENN producers [51,52].

ENN B1, like other emerging mycotoxins, is a natural unregulated contaminant in food and feed, and the tolerable daily intake and maximum levels in humans and animals remain unestablished, due to the lack of experimental data [29].

Exposure to ENN B1, even if it is a substance with favorable properties, is related to possible adverse effects in animals and humans [45,53,54,55].

Considering the toxicological aspects, it is already consolidated that ENN B1 in vitro exerts cytotoxic activity [30,36,56] and, moreover, impairs the cell cycle, induces oxidative stress and changes in mitochondrial membrane permeabilization, exerting negative genotoxic and estrogenic effects [57]. ENN B1 has effects at the mitochondrial level [37], causing an increase in ROS production, and intervenes in the modulation of some key mechanisms of inflammation [58,59].

ENN B1’s mechanism of action, which could be also multiple, is still unclear, but recently, it was demonstrated that is not a Ca^2+^ ionophore [52], even if in the past it was considered as such [55]. It induces alterations in Leydig cells affecting male reproductive system [60], possibly exhibits neuronal cytotoxicity and could be potentiating the effect of other mycotoxins on this cell line [18], and is a substrate of P-gp as demonstrated in Caco-2, Madine-Durbin Canine Kidney (MDCK) type II cells [61] and other human carcinoma cell lines [37]. In a Caco-2 serum-free model, even if permeability seems to not be affected, intercellular junctional organization seems to be perturbated, and an increase of IL-8 is also reported [62].

ENN B1 seems to affect the cholesterol biosynthesis pathway [63] as demonstrated in HeparG spheroids, a very interesting aspect strictly connected to the inhibition of the enzyme acyl-CoA:cholesterol acyltransferase [35]. Studies conducted on Daphnia magna with ENN B1 (0.25 μM and 1.5 μM) demonstrated negative effects at the individual level, inducing changes in body growth and sexual maturity [55].

An in vivo study in mice demonstrated an immunomodulatory effect of ENN B1 [29,64], and recent findings on porcine alveolar macrophages (PAMs) suggest a modulation of immune response and a potential to disrupt immune homeostasis [65]. ENN B1 showed a biphasic time- and dose-dependent effect: an initial immune activation inducing upregulation of pro- and anti-inflammatory cytokines (e.g., IL-1β, IL-6, IL-10, TNF-α), COX enzymes and the antioxidant gene GPX-2 and an increase in cell viability, while long term exposure induced a downregulation of these markers and reduced cell viability, indicating cytotoxic and immunosuppressive effects [66].

### 2.2. Beauvericin

Beauvericin, a cyclic hexadepsipeptide, is produced by several fungal genera, notably by *Fusarium* spp., which are important contaminants of crops used as foods or feeds, as well as major mycotoxin producers such as fumonisins and trichotecenes. This mycotoxin was first flagged by EFSA in 2014 as an emerging mycotoxin together with enniatins [25]. In this first opinion, EFSA highlighted the need for relevant in vivo toxicity data to perform a risk assessment for consumers or farm animals exposed to contaminated feeds. The scant data available at time pointed out rapid absorption and extensive metabolism in warm-blooded vertebrates, with low potential for bioaccumulation, and moderate acute toxicity in vivo [oral LD50 100 mg/kg b.w. in mice]. Beauvericin is cytotoxic in vitro by inducing several mechanisms, such as ionophoric properties, enzyme inhibition, oxidative stress and/or apoptosis, as confirmed by recent assays [67]. EFSA noted that a few in vitro studies suggested potential concerns deserving further investigation, such as clastogenicity in human lymphocytes and impaired differentiation of monocyte–macrophage cell lines [25,38,68].

Further studies ruled out a genotoxic potential in vivo, i.e., the main issue of concern in the EFSA’s assessment framework: new in vitro studies in mammalian cell lines as well as in vivo studies (comet assay and micronucleus) provided no convincing evidence for a direct effect on the induction of chromosomal damage or DNA strand breaks, although an indirect effect at high concentrations (e.g., from oxidative stress or apoptosis) might not be ruled out [69].

On the other hand, a project conducted under the umbrella of EFSA highlighted adverse effects on the reproductive, endocrine (thyroid) and immune systems in a repeated-dose toxicity assay in mice in vivo. Noticeably, effects showed some sex-specificity: the No Observed Adverse Effect Level (NOAEL) for female mice was 1 mg/kg b.w. per day (increased thyroid pycnotic nuclei and endometrial hyperplasia), while on male mice, it was 0.1 mg/kg b.w. per day, based on thyroid effects (reduced colloid and altered T4 serum levels) [66]. Beauvericin as an ionophoric agent can promote the intracellular uptake of cations; Ca^2+^ increases the resorption of thyroid colloid leading, as a feed-back mechanism, to upregulated glucose metabolism and protein synthesis in the gland [70]. This may be a plausible mechanism for the observed thyroid effect of the mycotoxin. Vacuolation of adrenal cortical cells in females is a further indicator of the endocrine-disrupting effect of beauvericin. The spleen as immune tissue is another sensitive target, as effects were observed down to the lowest dose level tested, 0.1 mg/kg bw, albeit the dose-response relationship was not clear: upregulated cytokines and increased white pulp in females and males, respectively [66]. In addition, more recent mechanistic studies have highlighted other targets of beauvericin toxicity, such as enteric glial cells [71], oocyte maturation and hormone production in the ovary [72,73]. The multiple targets of beauvericin, and in particular the relevance of immune and endocrine effects with identified points of departure at or below 0.1 mg/kg bw, call for a more systematic investigation of its toxicology being needed, as highlighted recently by Behr and colleagues [29]. The patterns of effects observed indicate possible sex- and life stage-driven susceptibility; thus, data on long-term and developmental effects as well as on early biomarkers are priority components to improve the hazard characterization.

## 3. ENNs and Beauvericin Occurrences

Enniatin B (ENN B) is currently the most widely analyzed mycotoxin, having been detected most frequently (alone or in combination with other mycotoxins) in unprocessed and processed cereals and grain-based products from European countries. The concentrations analyzed in cereals vary considerably, ranging from a few μg/kg to over mg/kg. When considering emerging mycotoxin mixtures, ENN B and B1 were the most frequently encountered when two mycotoxins were detected, while ENN B, B1 and A1 were the most frequently detected when three mycotoxins were present [9]. Regarding exposure risk, EFSA concluded that acute exposure to ENNs does not raise concerns for human health, but risk of chronic exposure could not be excluded, and experts are evaluating chronic exposure to carry out a proper risk assessment [74].

In a Norwegian study [75], samples of fish feed, vegetable meal, and vegetable oils used in the production of fish feed were analyzed to identify the presence of mycotoxins. ENN A was found at levels of 1% in vegetable meal, 0.5% in fish feed, and 10% in vegetable oils. Average ENN A values were found to be 20 ± 12 μg/kg in vegetable oils; however, ENN A contamination levels in vegetable meal and fish feed were below the Limit of Quantification (LOQ). ENN A1 showed prevalence levels of 7% in vegetable flours, 2% in fish feed, and 29% in vegetable oils. The average ENN A1 values detected were 56 ± 49 μg/kg in vegetable flours, 22 ± 7 μg/kg in vegetable oils, and 12 ± 27 μg/kg in fish feed.

Of all the matrices analyzed, ENN B was the most frequently detected mycotoxin, showing the highest prevalence levels (88% in vegetable oils, 80% in fish feed, and 15% in vegetable flours). ENN B was measured in concentrations ranging from 250 to 450 μg/kg in fish feed and vegetable oils, respectively.

The average ENN B value was 135 ± 186 μg/kg in vegetable meals, 114 ± 119 μg/kg in vegetable oils and 37 ± 35 μg/kg in fish feed. In contrast, the prevalence levels of ENN B1 were 10% in vegetable meals, 27% in fish feed and 65% in vegetable oils. Average ENN B1 values were found to be 78 ± 63 μg/kg in vegetable meals, 38 ± 27 μg/kg in vegetable oils and 18 ± 9 μg/kg in fish feed [75]. Following the analysis of 20 fish feed samples, ref. [76] found mean values of 0.9 μg/kg for ENN A, 1.77 μg/kg for ENN B1, 1.1 μg/kg for ENN A1 and 0.89 μg/kg for ENN B, with a prevalence of 100% [6].

In Europe, infant foods contain a median concentration of approximately 85 µg/kg of ENN A, 19.41 µg/kg of ENN A1, 41.86 µg/kg of ENNB and 31.58 µg/kg of ENN B1 [9].

The median concentration found in cereals was 29 µg/kg for ENN A, 14.82 µg/kg for ENN A1, 901.65 µg/kg for ENN B, and 819 µg/kg for ENN B1. In contrast, the mean concentrations were found to be 128.87 µg/kg for ENN A in cereal derivatives, 12.97 µg/kg for ENN A1, 152.47 µg/kg for ENN B, and 481.88 µg/kg for ENN B1.

In Spain, one study found ENN B average total concentrations of 1.0 ± 1.9 μg g^−1^ in pig feed [77], while another one characterized the occurrence in a total of 347 cereal-based products: the most contaminated food was bread rusks, followed by tin bread (average of 178.68 μg/kg and 163.32 μg/kg, respectively), whereas baby food showed the lowest contamination values (mean of 6.71 μg/kg). Sweet bakery products also showed between 11.68 and 23.42 μg/kg for muffins and cakes. The authors found lower levels of contamination in whole meal bread (74.46 μg/kg) than in white bread (103.5 μg/kg); this could be explained by considering that baking processes and the different origins of flours with varying levels of background contamination may affect the levels of ENN B found [78].

A recent Italian study assessed the levels of contamination by beauvericin and enniatin mycotoxins in baby food using chromatographic–mass spectrometric analysis, and ENN B was found to be present at a concentration of 11 μg/kg, with the highest incidence (90%) found in biscuits (average value: 4.1 μg/kg). The average concentrations of ENN B in cereal-based creams and pasta were 1.3 μg/kg and 0.77 μg/kg, respectively. ENN B1, on the other hand, was most prevalent (30%) in biscuits, with an average concentration of 1.5 μg/kg and a maximum concentration of 2.2 μg/kg, and the average concentrations of ENN B1 in samples of cereal-based creams and pasta were 2.3 and 1.3 μg/kg, respectively [79].

In Europe, baby food products appear to be, in general, the most contaminated commodity by ENNs, and in a study performed on 180 samples, 79 were positive for ENN B1 detected by LC-MS/MS.

ENNB1 was also the most prevalent ENN in maize silage, collected across 10 European countries [80].

In a Belgian study on wheat, set up also to highlight the influence of climate change and geographic location on *Fusarium* mycotoxin presence, ENN B1 was detected by Ultra-Performance Liquid Chromatography–Tandem Mass Spectrometry (UPLC-MS/MS) in 49 samples collected in 2024, with an average concentration of 185 µg/kg [81].

Furthermore, in a longitudinal study on eighty-seven consecutive breast milk samples obtained from new mothers residing in Austria and following a regular mixed diet, it was found that ENN A was present in trace amounts (below the LOQ of 1 ng/L) in 5% of the samples collected [82].

Already in 2014, EFSA performed a first overview of beauvericin (BEA) occurrence, based on the results of a call for data launched in 2010 as well as on established analytical methods for the parent compound, although not for possible metabolites [25]. Data on over 12,000 samples of food, feed and unprocessed grains were provided by 12 European countries, most of them fulfilling the required quality criteria. The proportions of samples with detectable levels were 20%, 21%, and 54% in food, feed, and unprocessed grains, respectively. For food, the highest mean concentrations were measured in dried fruits, followed by oilseeds and cereals for infants and children; for feed and unprocessed grains, the highest mean concentrations were measured in maize gluten. Physical processing (cleaning, milling) results in a reduction in the concentrations of beauvericin in the refined product with a concurrent increase in the cereal by-products; on the other hand, hot drying and ensiling procedures did not affect the concentrations. The chronic daily intake estimates ranged from 0.003 μg/kg b.w. to 0.050 μg/kg b.w. up to 0.093 μg/kg b.w. for the 95th percentile: overall, cereals and derived products featured as the main determinants of BEA exposure. Obviously, these estimates were considered with caution due to the many uncertainties (e.g., data from only a fraction of Member States) and, most important, the lack of toxicological data which prevented any risk assessment. Noticeably, the co-occurrence with ENNs is frequent, which is not unexpected, because BEA and ENNs are structurally related and are produced by *Fusarium* spp., as well as with other traditional mycotoxins. Synergic and antagonist effects are still under investigations, and this aspect requires further attention considering fusario-toxins.

No systematic, whole-diet, appraisals of dietary exposure have been attempted after; meanwhile, several recent papers provide up-to-date evidence of BEA occurrence in certain food groups/categories in individual countries [25].

A recent Italian paper investigated BEA in cereal-based infant foods (creams, biscuits, and pasta), where LOQ was founded to be 0.60 μg/kg. Concentrations above the LOQ were detected in 15% pasta samples and in 10% of creams and biscuits; maximum concentrations were 1.1, 0.2 and 0.41 μg/kg in pasta, biscuits and creams, respectively. Overall, BEA contamination of infant foods was significantly lower than ENNs, which was investigated in the same study [79].

BEA was also found in 100% samples of food supplements derived from green (unroasted) coffee beans at mean concentration of 5 μg/kg [83].

While a comprehensive appraisal of BEA occurrence is unfeasible, the available data indicate that contamination of plant-based foods and food ingredients is widespread, with grains and grain-based foods featuring prominently. Co-occurrence with ENNs is frequent, as they both are produced by *Fusarium* spp., but concentrations of BEA tend to be lower, e.g., in Irish oat. However, the measured values of BEA often cover a broad range, with levels in the upper range 5–10-fold higher than average/median values [84,85,86,87]. The reasons for such “peak” occurrences are not known; however, they might pose concern since BEA might elicit adverse effects also upon short-term exposure, e.g., on prenatal development.

BEA can also be a feed contaminant. The increasing use of more sustainable plant-based feed ingredients in aquaculture might make feed more liable to mycotoxin contamination: in particular, detectable levels of BEA can be found in more than 50% of main feed ingredients such as maize and soy beans: higher levels are found in maize (median as 90th percentile 7.7 and above 100 μg/kg, respectively) [88]. A Spanish study found BEA in 14% of aquaculture feed samples in Spain (mean concentration 30 μg/kg), but no carry-over was observed to gilthead sea bream and Atlantic salmon, in accordance with the low bioaccumulation potential of the mycotoxin [76]. Another Spanish study found a widespread contamination of pig feeds, while carry-over, as measured by urine biomonitoring, was very low [77]. The available data suggest that BEA contamination of feeds has no significant impact on the safety of foods of animal origin, while it might pose a risk to animal health due to the high mycotoxin toxicity. Table 2 summarizes ENNs and BEA occurrences.

## 4. Climate Change Influence

By 2050, in the north of Europe, milder and more humid climate is expected, influencing *Fusarium* growth and affecting their geographical distribution [4]. Compared to other continents, Europe is warming much faster, with an average rate of about 0.5 °C per decade in the period 1991–2021 [90].

The incidence of heat waves, compared to cold spells, has increased. In addition, extreme precipitation has also increased in recent decades, particularly in northern and eastern Europe, unlike in the Mediterranean region, where more frequent and severe droughts are observed [89,91].

Several scientists have characterized and studied the influence of weather conditions on the appearance of mycotoxins in crops, particularly corn and wheat. These cereals are widely used in Europe but are among the crops most susceptible to mycotoxin contamination, especially when adverse weather conditions occur. Numerous scientific studies have examined how weather patterns dictate the prevalence of mycotoxins in maize and wheat. In Europe, maize is typically grown during the spring and summer, whereas wheat follows a longer cycle from late autumn to early summer.

Heavy rainfall during late spring creates an environment conducive to *Fusarium* species in both crops, leading to elevated levels of mycotoxins—most notably DON, followed by ZEN and (in maize) FUMs. Conversely, hot and dry summer conditions favor Aspergillus species, increasing the frequency of aflatoxin contamination in maize. In parts of Southern Europe, extreme heat is already shifting agricultural ecosystems. Because maize and wheat are essential staples for both food and animal feed, these contamination trends pose a significant threat to the safety of the entire supply chain [92].

**Figure 2:**
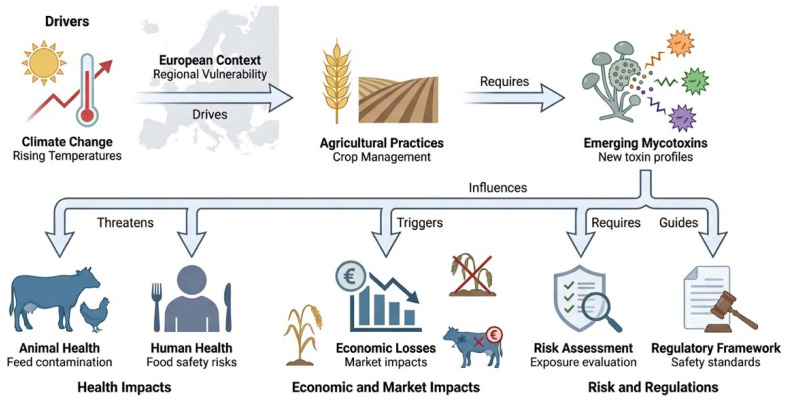
Interconnected effects of climate change on emerging mycotoxins in Europe, highlighting climate factors, agricultural changes, new mycotoxin profiles, risks to health, economic impacts, and regulatory measures (by Figurlabs).

Despite Europe’s strict regulations on mycotoxins in food and feed, and its commitment to preventing and controlling them, mycotoxin contamination in various food and feed products is a growing concern. In fact, climate change has led to extreme conditions, which means crops are more likely to be contaminated by mold and the mycotoxin content in food has increased a lot; therefore, the presence of mycotoxins in food and feed poses a significant risk to public health and animal welfare [1,93] (Figure 2).

It has been shown that the production of mycotoxins is strongly influenced by environmental conditions such as increased carbon dioxide levels, rising ambient temperatures, and alternating periods of extreme drought and extreme precipitation that vary on an annual basis [94,95,96] and by additional factors, including the type of substrate, nutrient availability, substrate percentage, environmental humidity, the presence of other molds and competition with other microorganisms [96,97,98]. Additionally, cultivation procedures influence the presence of harmful agents such as insects, which damage crops and promote mycotoxin contamination. Based on current data, it is foreseeable that climate change will have a significant impact on the production of toxicogenic molds in the future and, consequently, on the prevalence, frequency of occurrence, and types of mycotoxins, posing a serious risk to health and the economy as well as a threat to global food security [99].

Traditional mycotoxins, such as aflatoxins, and emerging mycotoxins from the *Fusarium* genus, including enniatins (ENNs) and beauvericin (BEA), are expected to become increasingly problematic in Europe as *Fusarium* species spread northwards. BEA is also a *Fusarium* metabolite, and it can be extensively metabolized in plants relevant to modified BEA by-products; therefore, there is significant uncertainty for exposure assessment. In contrast, Aspergillus species will be concentrated in southern regions. Concerns about contamination of cereal-derived products such as flour, pasta, and couscous are growing, as ENNs are unregulated contaminants and Europe accounts for more than half of global contamination. Furthermore, ENNs are resistant to common food processing methods, including boiling, baking, and frying. Their lipophilic properties allow them to uptake cell membranes and perturb the cationic cell balance. Although their cytotoxicity has been demonstrated in vitro, the available in vivo toxicological data are insufficient for a comprehensive risk assessment. In addition to cereals, the risk of mycotoxins is increasing in aquaculture through alternative protein sources, raising concerns about human exposure through fish consumption. Effective drying and storage practices, as well as moisture control, remain key prevention strategies. Proactive regulatory and monitoring measures are urgently needed to address these emerging risks in the context of a rapidly changing climate, representing a still unresolved issue in risk assessment.

## 5. Recommendation for Risk Reduction

Mycotoxin contamination in food and feed has led to increased production costs and reduced animal performance and profitability, causing particular concern among farmers.

To prevent or minimize mold growth and mycotoxin contamination in cereals, effective drying and storage are essential [100]. For example, to ensure stable medium-term storage of temperate cereals without deterioration or post-harvest mycotoxin contamination, it is important to ensure that the moisture content does not exceed 14.5–15% (wet weight = 0.70 water activity, aw). EU legislation sets out guidelines for B-type trichothecene contamination (deoxynivalenol, DON) and zearalenone (ZEN) in wheat and barley. Unfortunately, there is less information on secondary metabolites that may be present due to fungal colonization and differences in production [101].

As already reported, many factors, from the temperature to the humidity, can influence mold and mycotoxin production, highlighting the need for proactive measures to address emerging mycotoxin risks in a changing climate [9].

## 6. Conclusions

The landscape of mycotoxin contamination in food and feed is changing because of climate change, creating new challenges for global food safety. Fungal infections and the emergence of new mycotoxins are indeed driven by rising temperatures and extreme weather events that increase plant stress. In fact, in Southern Europe, extremely hot summers have already led to changes in agricultural ecosystems. All of the effects mentioned are a cause of great concern, as corn and wheat are staple foods and ingredients in animal feed, and their contamination poses an additional problem for food and feed chains. The issue of mycotoxins and the contamination of raw materials, food and feed have been well documented, and growing interest is related to climate change and potential contamination scenarios by mycotoxin-producing fungi in Europe. This aspect also concerns mycotoxins produced by *Fusarium* spp., both traditional and so-called emerging ones.

While there are well-defined legislative limits for traditional mycotoxins, the situation is different for emerging ones. These are currently unregulated and not always analytically searched, but they have not only been detected in various raw materials and products in Southern Europe, particularly Italy and Spain, but a different trend is expected in the coming years, with an increase in Northern Europe.

The possibility of synergistic and additive effects not only between emerging mycotoxins but also in association with mycotoxins such as fumonisins or deoxynivalenol, and other trichothecenes, represents another issue to take into consideration.

In the case of a number of emerging mycotoxins, the available data are often insufficient for a sound risk assessment, yet they are sufficient to raise concern for the safety of foods and feeds as well as to trigger the need for further study.

## 7. Methods

Inclusion criteria included peer-reviewed journal articles in English as well as relevant scientific documents issued by authoritative institutions, including EFSA scientific opinions, reports, and guidance documents. These sources were included due to their use in risk assessment and regulatory decision-making within the European Union.

Exclusion criteria included non-English publications, conference abstracts, editorials, and studies with insufficient methodological information.

Since the term “emerging mycotoxins” has been used in the scientific literature since the early 2000s and more widely since around 2010 in relation to advances in analytical methods that have made it possible to detect a broader range of fungal metabolites, the literature search was conducted for studies published between 2010 and 2026. Detailed searches of relevant pubblications on different databases, including MEDLINE/PubMed, Scopus and Web of Science, using the keywords “emerging mycotoxins” OR “enniatins” OR “enniatin A1” OR “enniatin A2” OR “enniatin B” OR “enniatin B1” OR “beauvericin”, with the combinations within the reported terms and “Europe”, “climate changes”, “occurrence”, and “toxicity”, were performed. The quality and relevance of the included studies were evaluated based on their study design, methodological robustness, and relevance to the research topic.

## Figures and Tables

**Table 1:** Main differences between enniatins and beauvericin.

Characteristic	Enniatins	Beauvericin
Type of amino acids	N-methyl-valine, N-methyl-isoleucine, or leucine (aliphatic)	N-methyl-phenylalanine (aromatic)
Main producers	*Fusarium* spp.	*Fusarium* spp., *Beauveria bassiana*
Biological activities	Antibiotic, ionophoric	Antibacterial, insecticidal, cytotoxic
General formula	Alternation of D-Hiv and N-Me-Val/Ile/Leu	Alternation of D-Hiv and N-Me-Phe

**Table 2:** Occurrence of ENNs and BEA.

Emerging Mycotoxin	Matrix	µg/kg	Country	Ref.	Year
ENN A	infant foods	85	Europe	[9]	2025
	cereals	29	Europe	[9]	2025
	derived from cereals	128.87	Europe	[9]	2025
	plant-based oils	20 ± 12	Norway	[75]	2023
	fish feed	0.9	Spain	[89]	2022
ENN A1	infant foods	19.41	Europe	[9]	2025
		6.71	Spain	[78]	2023
	cereals	14,822.61	Europe	[9]	2025
	derived from cereals	12,974.59	Europe	[9]	2025
	plant-based meals	56 ± 49	Norway	[75]	2023
	plant-based oils	22 ± 7	Norway	[75]	2023
	fish feed	12 ± 27	Norway	[75]	2023
		1.1	Spain	[76]	2022
ENN B	infant foods	41.86	Europe	[9]	2025
		6.71	Spain	[78]	2023
	cereals	901.65	Europe	[9]	2025
	derived from cereals	152.47	Europe	[9]	2025
	cereal-based creams	1.3	Italy	[79]	2025
	biscuits	4.1	Italy	[79]	2025
	pasta	0.77	Italy	[79]	2025
	bread rusks	178.68	Spain	[7]	2018
	tin bread	163.32	Spain	[78]	2023
	Whole meal bread	74.46	Spain	[78]	2023
	white bread	103.5	Spain	[78]	2023
	plant-based meals	135 ± 186	Norway	[75]	2023
	plant-based oils	114 ± 119	Norway	[75]	2023
	fish feed	37 ± 35 0.89	Norway Spain	[75] [76]	2023 2022
	pig feed	0.001 ± 0.0019	Spain	[77]	2022
ENN B1	infant foods	31.58	Europe	[9]	2025
	cereals	819	Europe	[9]	2025
	derived from cereals	481.88	Europe	[9]	2025
	cereal-based creams	2.3	Italy	[79]	2025
	biscuits	1.5	Italy	[79]	2025
	pasta	1.3	Italy	[79]	2025
	wheat	185	Belgium	[81]	2025
	plant-based meals	78 ± 63	Norway	[75]	2023
	plant-based oils	38 ± 27	Norway	[75]	2023
	fish feed	18 ± 9 1.77	Norway Spain	[75] [76]	2023 2022
BEAUVERICIN	infant foods	0.60	Italy	[79]	2025
	food supplements from green coffee beans	5	Europe	[82]	2025
	aquaculture feed	30	Spain	[87]	2025
	pig feed	0.03	Spain	[77]	2025

## Data Availability

No new data were created or analyzed in this study.
